# Study on the Elimination Method of Wind Field Influence in Retrieving a Sea Surface Current Field

**DOI:** 10.3390/s22228781

**Published:** 2022-11-14

**Authors:** Xinzhe Yuan, Jian Wang, Bing Han, Xiaoqing Wang

**Affiliations:** 1 National Satellite Ocean Application Service, Beijing 100081, China; 2 Key Laboratory of Space Ocean Remote Sensing and Application, Ministry of Natural Resource of the People’s Republic of China, Beijing 100081, China; 3 School of Systems Science and Engineering, Sun Yat-sen University, Guangzhou 528406, China; 4 Aerospace Information Research Institute, Chinese Academy of Sciences, Beijing 100094, China; 5 School of Electronic and Communication Engineering, Sun Yat-sen University, Shenzhen 518052, China

**Keywords:** sea surface current, along-track interferometric SAR, Gaofen-3 satellite, CDOP model

## Abstract

An along-the-track interferometric synthetic aperture radar (ATI-SAR) system can estimate the radial velocity of a moving target on the ground and on a sea surface current. This acquires the interference phase by combining two composite SAR images obtained by two antennas spatially separated along the direction of movement of the platform. The key to retrieving the sea surface current is to remove the interference of sea surface waves, wind-generated current, and Bragg phase velocity in the interference Doppler velocity. Previous methods removed the surface waves, Bragg phase velocity, and other interferences based on externally-assisted wind fields (e.g., ECMWF), using the M4S or other models. However, the wind fields obtained from ECMWF and other external information are often average results of a large temporal and spatial scale, while the images obtained from SAR are high-resolution images of sea surface transients, which are quite different in time and space. This paper takes the SAR image data of the Gaofen-3 satellite as the research object and employs an SAR-based wind field retrieval method to obtain an SAR-observed transient wind field. Combined with the CDOP model, the interference of Doppler velocities, such as the sea surface wave, wind-generated current, and Bragg wave phase velocity, was calculated and subtracted from the Doppler velocity, to obtain the sea surface velocity result. Then, the current field measured by the shore-based HF radar was compared with that obtained by correcting the ATI Doppler velocity based on the SAR retrieved wind field and the ECMWF wind field. The comparison of results indicated that the wind field correction result based on the SAR retrieved wind field was closer to the current field measured by the shore-based HF radar than the wind field correction result based on the ECMWF wind field.

## 1. Introduction

Ocean surface current is an important research field and plays an important role in climate change, oceanic engineering, fishery resources, energy and oceanic ecosystem, etc. [1]. Therefore, the observation of oceanic surface flow is significant. In coastal and continental shelf areas, sensors such as altimeters and scatterometers are of limited use for small-scale physical phenomena [2]. Synthetic aperture radar (SAR) has been widely used to observe oceanic surface currents, owing to its full-time and all-weather capabilities and high spatial resolution. In addition, the data received by SAR can be processed by algorithms. However, it is still challenging to accurately retrieve the surface current from SAR image data. It is critical to obtain the orbit parameters of the satellite, the wind field of the study area, and other information. Due to the differences in observation time and system, satellite observation is rarely compared with marine field observation data. Therefore, most research on space-borne SAR concerns ocean interpretation and qualitative comparison of ocean currents to verify its ability to detect various ocean physical phenomena [3] (such as ocean fronts, eddies, internal waves, etc.).

Currently, two techniques can be adopted to achieve current field inversion based on SAR: doppler centroid analysis (DCA) and along-track interferometric (ATI). Both can obtain the radial velocity in the current field. The DCA-based currents are less accurate than the ATI-based ones, but close to short-baseline ATI can produce quality results; meanwhile, DCA is an alternative to divided-antenna mode ATI, and the results demonstrate the true potential of the ATI technique at near-optimum baselines [4]. Along-track interferometric synthetic aperture radar (ATI-SAR) has the unique ability to measure sea surface velocity. In 1987, Goldstein and Zebker [5] of NASA’s Jet Propulsion Laboratory first proposed the method of using ATI to measure high-resolution sea currents, and its feasibility was verified through experiments. Goldstein et al. [6,7] used two L-band radar antennas to investigate the measurement of tidal current at the outlet of the Gulf, and two antennas were installed on an aircraft. This was the first feasibility study. In addition, the ability of ATI-SAR to measure surface velocity was demonstrated by Thompson, Graber, and others through several studies with airborne radar. The fundamental concept for measuring surface velocity is that the phases of two complex SAR images obtained from the same scene and the same antenna have a short time delay, and the phase difference is proportional to the line-of-sight velocity of the target [8,9]. The theoretical background and numerical model of ATI-SAR are presented in [8]. Meanwhile, many experiments on different platforms have been conducted to validate ATI technologies, such as airborne, space shuttle, and satellite [9,10,11,12]. The first study based on bistatic TanDEM-X (TDX) was reported in [13]. For two SAR acquisitions of the same area on the ground, at two times separated by a few milliseconds, the pixel-by-pixel phase difference between the two complex focused SAR images is related to the line-of-sight (LOS) velocity of the imaged surface block, also known as the radial velocity. ATI-SAR can be realized using two antennas installed on the same platform. Additionally, experiments and studies on the applicability of the satellite formation system are presented in [14]. The results indicated that the satellite formation has a long baseline, close to the theoretical optimization analysis, and it has advantages in current measurement. Besides the experimental systems described above, there are no operational civilian satellites with ATI capability. To verify ATI technology and provide support for future satellites, domestic ATI sea surface current measurement experiments were conducted for the first time based on China’s Gaofen-3 satellite (GF−3) [15] from 2018 to 2019. Yuan et al. [16] proposed using ATI technology to retrieve the ocean surface current field.

The Doppler velocity obtained using ATI technology includes the contributions of the current field and the images of various wind-generated currents, the Bragg wave phase velocity, and the orbital velocity. Only by removing these effects can the current field be accurately obtained. The process of removing the effect of the wind field is similar to that of removing the effect of the wind field using Doppler anomaly. Considering the scattering contribution of large-scale waves and Bragg waves in the ocean, these ocean surface wind fields play a decisive role. Compared with the wind field retrieved from SAR images, the ECMWF wind field has errors, due to its large spatial grid, and the root SAR images do not match well.

The above research ignored the influence of different ocean wind fields on ocean surface current field retrieval. In this paper, an SAR image of the Gaofen-3 satellite interference mode was used. First, the SAR image was preprocessed using radial velocity retrieval. Then, different wind fields were input into the CDOP model, to obtain the retrieved radial velocity, which was quantitatively compared with the radial velocity obtained by a shore-based radar. To investigate the influence of different wind fields, this paper compared the parameters of two wind fields input into the CDOP model (one was based on SAR image retrieval and the other was reanalyzed from ECMWF), and the ocean surface current field was finally retrieved. The results show that the wind field correction result based on the SAR-retrieved wind field was closer to the current field measured by the shore-based HF radar than the wind field correction result based on ECMWF.

## 2. Doppler Center Frequency Retrieval Method

### 2.1. Components of Doppler Frequency

For the ATI-SAR, the interference phase of the images corresponding to two antennas after complex multiplications is as follows:

(1) $$\phi =-\frac{2\pi }{\lambda }\cdot 2\cdot \Delta R=-\frac{4\pi Bv_{r}}{\lambda v_{p}},$$

where ΔR is the range difference of the same position, B is the baseline length of the satellite, $v_{r}$ is the radial velocity, λ is the wavelength of the radar relative to the operating frequency, and $v_{p}$ is the station velocity.

The radial velocity can be expressed as:

(2) $$v_{r}=-\frac{\lambda v_{p}}{4\pi B}\phi ,$$

Then, the Doppler frequency can be retrieved following the principles of ATI-SAR:

(3) $$f_{dc}=\frac{2v_{r}}{\lambda },$$

The Doppler frequency retrieved using ATI-SAR from the single-look complex data consists of several items [17]:

(4) $$f_{dc}=f_{dc}^{phys}+f_{dc}^{geo}+f_{dc}^{ele}+\Delta f_{dc},$$

where $f_{dc}^{phys}$ is the geophysical term, $f_{dc}^{geo}$ is the geometric attitude term, $f_{dc}^{ele}$ is the antenna electronic miss-pointing, and $\Delta f_{dc}$ is the residual error from an imperfect prediction of the non-geophysical terms or other unknown biases. For the ATI-SAR, the effect of $f_{dc}^{ele}$ on the overall result is negligible. For a stable satellite orbit and attitude, the measured Doppler shift $\Delta f_{dc}$ can be estimated from the SLC data or processed data. Generally, the residual variation after recalibration is about 3.8 Hz. The attitude is pre-corrected by $f_{dc}^{\mathrm{geo}}$ when imaging the satellite SAR RAW data. $f_{dc}^{phys}$ includes the contributions of various wind-generated currents in the current field, the Bragg wave phase velocity, and the orbital velocity.

### 2.2. Eliminating the Influence Algorithm and Model

The model function is configured with ASAR Doppler measurements and ECMWF winds, which correlate the Doppler shift and wind field based on the C-band radar configuration. The model is expressed as follows [16]:

(5) $$f^{DA}=CDOP(\phi ,u_{10},\theta ,pol),$$

where $u_{10}$ denotes the wind speed at a height of 10 m, ϕ is the angle between the direction of the SAR antenna and the wind direction, θ is the incidence angle, and pol is the polarization of the radar.

In this paper, to retrieve the Doppler shift caused by wind, the retrieved wind from the SAR (SLC image) is used to denote the parameters for the CDOP model function. Then, the $f_{b}$ caused by Bragg waves and $f_{ls}$ caused by large-scale waves are regarded as wind field modulation results. Thus, $f^{DA}$ can be approximated as:

(6) $$f^{DA}\approx f_{ls}+f_{b},$$

Therefore, processing of the Doppler anomaly can be derived as:

(7) $$f_{c}=f_{{}_{dc}}^{phs}-f^{DA},$$

For an easier geophysical interpretation, the Doppler shifts are converted to the surface radial velocity ($V_{c}$) using the following relation:

(8) $$V_{c}=-\frac{\lambda f_{c}}{2\sin\theta },$$

## 3. Situation of Data Acquisition

The GaoFen-3 satellite sea current measurement experiments were organized by the National Satellite Ocean Application Service (NSOAS), and the experiments were conducted in Qing Dao on 29 January 2022. The radar parameters were specially set by the Institute of Remote Sensing Satellite (IRSS, China Association for Science and Technology (CAST)). For the GF−3 satellite, the ATI mode was taken as the experimental mode. The radar echo imaging process was completed by NSOAS based on a modified GF−3 data processor. The HFSWR data of Qing Dao and sea current meter data in Beibu Gulf were provided by the North China Sea Marine Forecasting Center, Ministry of Natural Resources (NCSFC, MNR) and Beihai Marine Environment Monitoring Center Station, Ministry of Natural Resources (BHMEMC, MNR). Sun Yat-sen University, as one of the participating institutions in the project, worked with the satellite center to retrieve the sea surface current field.

To further verify the accuracy of correcting the Doppler current field based on SAR wind field retrieval and ECMWF wind field retrieval, an experiment was conducted on 29 January 2022 in Qing Dao, Shandong Province, China. Five images were obtained in the experiment. The specific times and regions of the observations are presented in Table 1.

**Figure 1:**
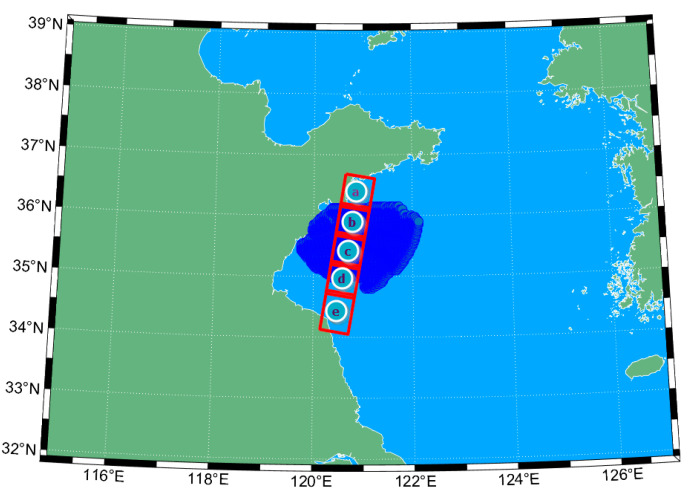
GF-3 satellite observation area (red) and HFSWR measurement area (blue) in the experiment conducted in Qing Dao in 2022.

Figure 1 shows the observation area in the yellow sea of China, near Qing Dao. The area observed by GaoFen-3 is within the red rectangle. There are five satellite observation areas, with a latitude and longitude ranging from 120.48° E to 120.88° E and 34.41° N to 36.38° N, namely, regions (a), (b), (c), (d), and (e), corresponding to the index in Table 1. Meanwhile, the HFSWR observing area is in the blue area. This paper chose regions (b) and (c), which can cover the satellite observation area, to compare the retrieved sea surface current of the SAR images.

## 4. Experimental Data Set

### 4.1. GF-3 Satellite ATI Experimental Mode

The 1 m resolution multi-polarization SAR satellites 01 and 02, also known as GaoFen-3 B and C (hereinafter referred to as GF−3B and GF−3C), are two follow-up operational satellites of China’s first polarization SAR−GF−3 satellite. The mission of the two satellites is to provide quantitative and stable remote sensing data for marine targets, marine dynamic environmental factor monitoring, and for emergency management, land, geology, water conservancy, environmental protection, agriculture, meteorology, and other applications through in-orbit operation of the GF−3 satellite network.

The GF−3B and GF−3C satellites inherited the mature technologies of the GF−3 satellites, except for the multi-polarization SAR and dual-frequency GPS. Compared with the GF−3 satellite, the two satellites have received system optimization and improvements, while preserving the main technical state. Specifically, the Automatic Identification System (AIS) signal-receiving system was added, to improve the monitoring capability for the main offshore monitoring target: offshore ships; the traditional scanning mode was changed to the TOPSAR mode; the spatial resolution and observation breadth of the wave mode were enhanced; the daily observation time was increased, to further improve the efficiency and availability of satellite usage; and the satellites carry an onboard real-time processor. Functions such as imaging processing, region of interest extraction, and target detection can be realized on the satellite, to enhance the capabilities for early detection, early prevention, and early response to maritime emergencies and marine and land natural disasters.

**Figure 2:**
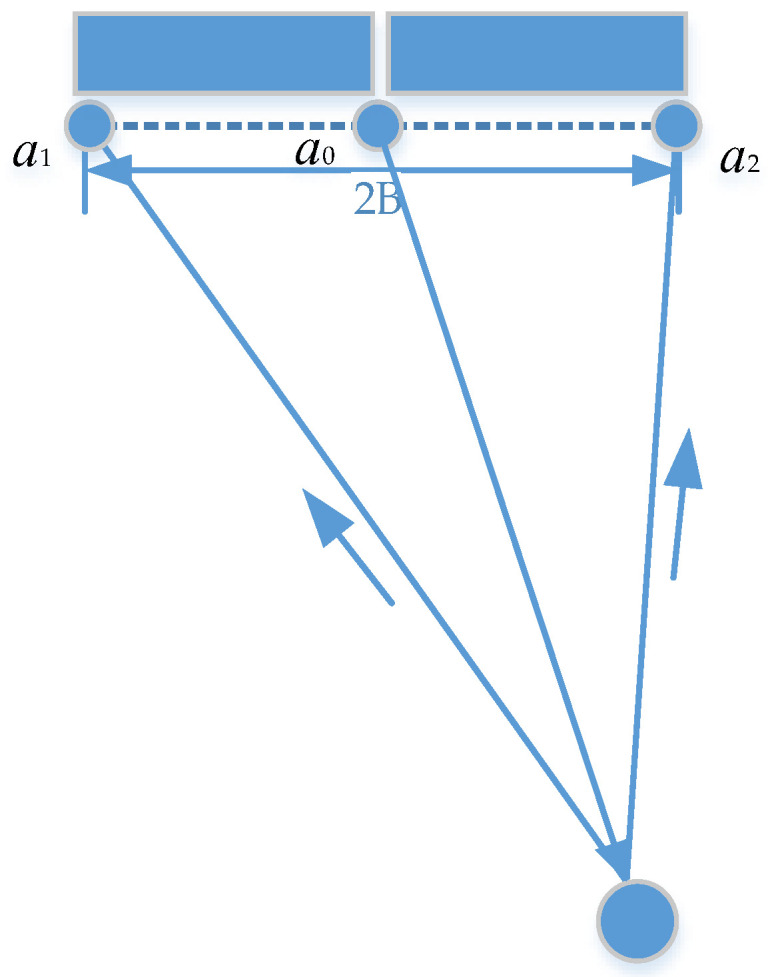
The schematic diagram of the GF−3 satellite SAR-ATI mode.

The ATI mode of the GF−3 satellite utilizes a similar principle to the DRA mode of the TerraSAR-X satellite [4], where the full aperture sends a signal, and the two sub-apertures simultaneously receive the echo. As shown in Figure 2, the full aperture phase center is at $a_{0}$, and the range between $a_{1}$ and $a_{2}$ is 2B. The signal received by apertures 1 and 2 can form an interferometric baseline in azimuth, with a length of B=3.75 m [18]. The radar parameters of the GF−3 satellite ATI mode are shown in Table 2.

### 4.2. Image-Related Parameters

The echo data received by the GF−3 radar are processed by the chirp scaling algorithm [17]. For this experiment mode, the data of the system in two channels can produce two SAR images (single-look complex images), which are the level-1 products. The SAR image parameters in the experimental mode are presented in Table 3.

### 4.3. HFSWR Data

The SeaSonde HF radar system can obtain wide-area ocean observations. The experiment data are acquired by the “COADS Seasonde” HF radar, which can continuously measure current. The HFSWR operational frequency is 24.5 MHz. The resolution of the measurements ranges from 500 m to 3 km, and the spatial angular resolution ranges from 1° to 5°. The RMSE of the measured radial velocity RMSE is lower than 7 cm/s.

In this experiment, the HFSWR data were recorded at 21:00 (UTC), 29 January 2022 on the coast of Qingdao. The range of the measured area was from 119.25° E, 34.89° N to 122.86° E, 36.10° N. Note that the HFSWR product data can generate due east and due north ocean flow field velocity data with latitude and longitude correspondence. Therefore, for the following comparison, it was necessary to convert the flow field velocity in both directions of the HFSWR data to the radial velocity of the SAR image, which is explained in detail in Section 5.3.

### 4.4. Input Wind Field

#### 4.4.1. Retrieved Wind Field from SAR Images

**Figure 3:**
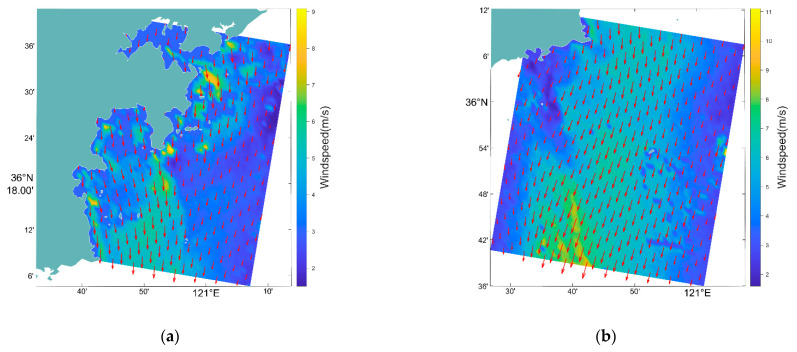

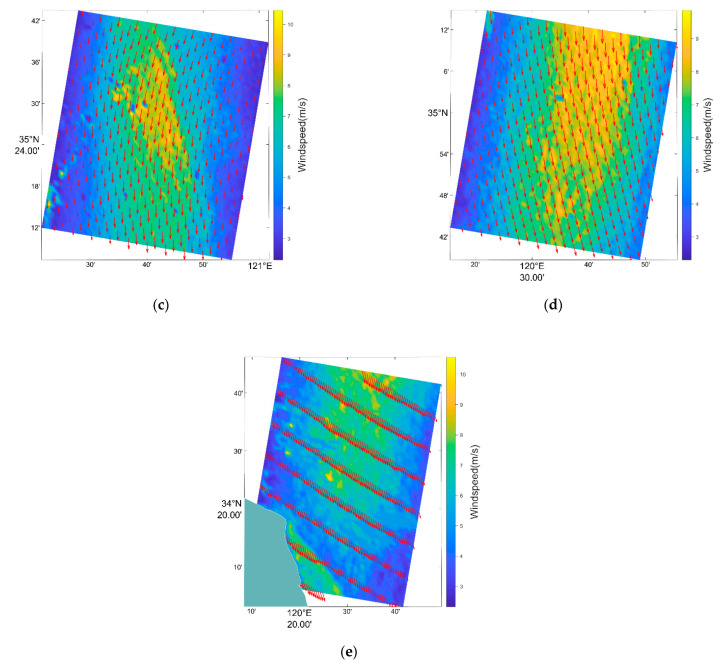
The input wind field of the five scenes. (**a**–**e**) are the wind fields in red square area of Figure 1.

To ensure the accuracy of ocean surface current retrieval, the wind field input is the wind field retrieved from the SAR radiation correction image. The sea surface wind field was retrieved using the CMOD-7 geophysical model function [19], combined with the incident angle, azimuth angle, and background wind direction observed using the SAR satellite. The wind field retrieval results of the above five SAR images are shown in Figure 3.

#### 4.4.2. ECMWF Reanalysis Wind Field

**Figure 4:**
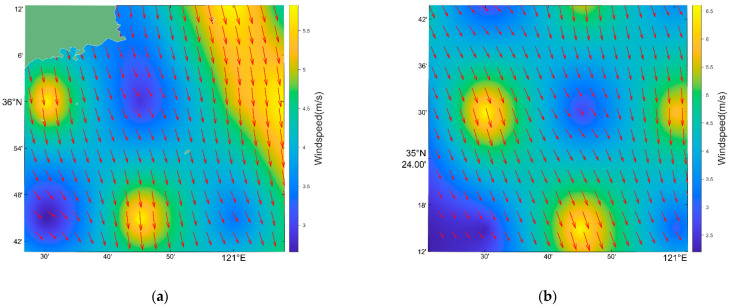
(**a**) The ECMWF reanalysis wind field of region (**b**) in the Figure 1. The ECMWF reanalysis wind field of region c in the Figure 1.

ERA5 is the fifth-generation of ECMWF reanalysis of global climate and weather over the past four to seven decades. Currently, the data are available from 1950, with climate data store entries for 1950–1978 (preliminary back extension) and from 1959 onwards (final release plus timely updates, this page) [20]. In the experiments, the European Centre for Medium Weather Forecasts (ECMWF) reanalysis wind field (ERA3) was applied, because there was no on-site wind measurement instrument. Its time resolution was 6 h, and the spatial resolution was 0.25° (about 25 km) [17]. The SAR images were re-analyzed at similar times, at 21:00 and 22:00, by selecting the latitude and longitude range of images (b) and (c), to interpolate to the accurate SAR image time. The results are shown in Figure 4.

## 5. Data Processing

Two types of data need to be processed before current extraction: the SLC image pairs generated by the GF−3 data processor, and the ECMWF wind field. The wind field generated in the processing is used as input for the CDOP mode.

**Figure 5:**
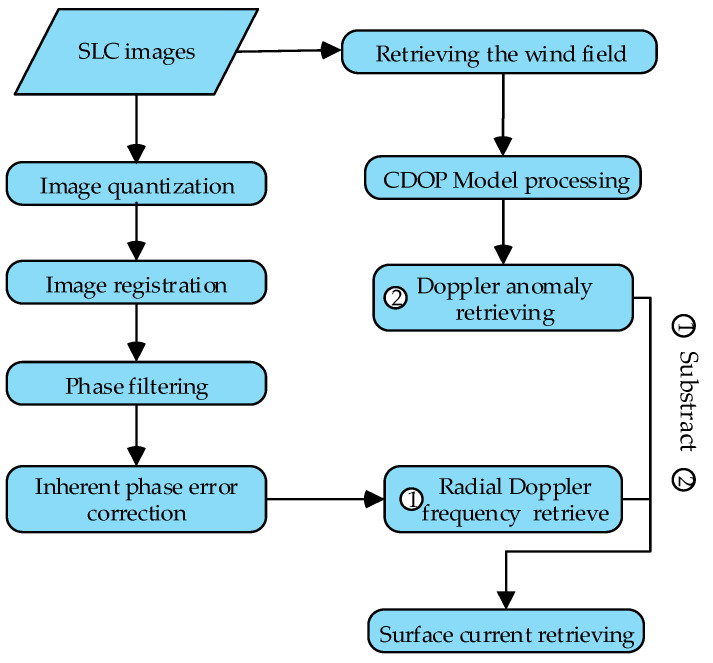
Data processing flow.

The processing steps are illustrated in Figure 5, and the details are given in the following sections.

### 5.1. Image Quantization

Image quantization is the process of converting the continuous transform intervals of the corresponding brightness of the image pixel points into a single value, i.e., the amplitude values of the spatial coordinates of the original gray image are discretized.

For the GF−3 experimental mode, the SLC image quantization is performed based on Equation (9).

(9) $$s=DN_{i}/32767\cdot QV+j\cdot DN_{q}/32767\cdot QV,$$

where s is the image quantization result; i and q denote channel I and Q, respectively; and *j* is the imaginary unit. DN is the channel data of the SLC image, and QV denotes the quality value of different channels.

### 5.2. Image Registration

For the ATI-SAR system, due to the existence of the along-track baseline, the pixels in the front and rear SLCs do not completely correspond, and there are several azimuthal pixel offsets for the position of the same target in the front and rear SLCs. Therefore, accurate registration of SLCs is necessary, to obtain effective ATI phases, and it is generally required that the registration accuracy should reach the sub-pixel level [4], otherwise the interference phases will be mismatched, which has a serious impact on retrieving the sea surface current field. In this paper, the correlation coefficient method [21] was adopted for SAR image registration. The registration processing is shown in Equations (10) and (11).

(10) $$s_{azi}=IFFT\left(FFT\left(s_{a}\right)\cdot \exp\left(-j\cdot 2\pi \cdot f_{d}\cdot \frac{\Delta d}{v_{s}}\right)\right),$$

(11) $$s_{ar}=IFFT\left(FFT\left(s_{azi}\right)\cdot \exp\left(-j\cdot 2\pi \cdot f_{r}\cdot \frac{\Delta R}{v_{c}}\right)\right),$$

where $s_{a}$ is the rear SLC image data, $s_{azi}$ is the azimuth registration result, $s_{ar}$ is the range registration result after the azimuth registration is completed. FFT signifies fast Fourier transformation in the azimuth direction in Equation (10). In Equation (11), FFT is in the range direction. IFFT means inverse fast Fourier transformation, and the transformation direction is the same as in the FFT operation. $f_{d}$ is the Doppler frequency, ranging from −PRF/2 to PRF/2, and $f_{r}$ is the range frequency ranging from −B/2 to B/2 (B is the bandwidth of the radar system). $v_{s}$ is the satellite velocity, and $v_{c}$ is the light speed. Δd is the azimuth distance of the two channels. ΔR is the range distance of the two channels. j is the imaginary unit.

After image registration processing, the sliding window method [15] is employed for processing data, and phase filtering is performed to obtain the neighborhood average. In this study, the window size was 64 × 64 pixels. For ATI-SAR, inherent phase error correction [20] is a vital step in the procedure. The phase errors are corrected every 64x64 pixels along the range direction. Then, these phase errors are eliminated, to complete the phase error correction.

### 5.3. HFSWR Data Processing

Since the measurement range of the HFSWR data is not necessarily within the observation range of the satellite radar, the HFSWR data need to be interpolated into the SAR image area, according to the latitude and longitude of the SAR image. It can be seen from Figure 2 that scene 1 and scene 2 cover the largest HFSWR measurement area. Therefore, only the HFSWR data of these two scenes were processed. The HFSWR data has four vital parameters, namely longitude, latitude, U, and V. According to the user manual, U is the east-direction current speed, and V is the north-direction current speed. Since the velocity of our retrieval was in the radial direction, it was then converted to the surface direction, and the current field also needed to be converted in this way. The range direction is shown in the following:

(12) $$\vec{Range}=norm(\vec{P_{image}})\times norm(\vec{P_{sat}}),$$

where $\vec{P_{image}}$ is the azimuth vector of SAR imaging, which can be obtained from the initial position of satellite imaging. $\vec{P_{sat}}$ is a vector from the center of the earth pointing to the center of the satellite trajectory. [×] represents the cross-product, and [norm] represents a normalized operation.

Thus, *U* and *V* can be converted to the ocean surface current field in the SAR image range direction:

(13) $$\vec{V\_current}=\vec{U}*\vec{Range}+\vec{V}*\vec{Range},$$

where [∗] represents the dot product of vectors.

**Figure 6:**
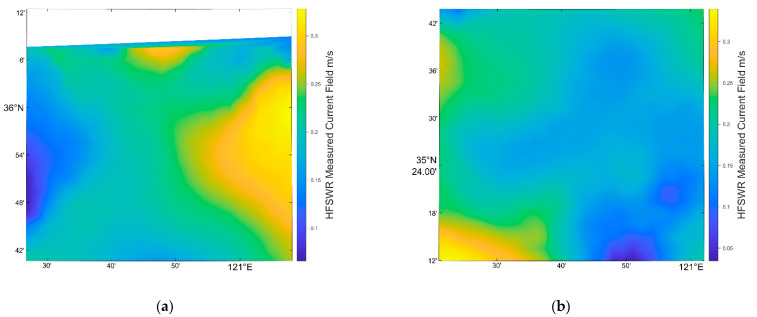
(**a**) The result of HFSWR data processing of scene b in Figure 1, (**b**) the result of HFSWR data processing of scene c in Figure 1.

Utilizing Formulas (12) and (13), the result of HFSWR data processing is shown in Figure 6.

## 6. Results and Analysis

**Figure 7:**
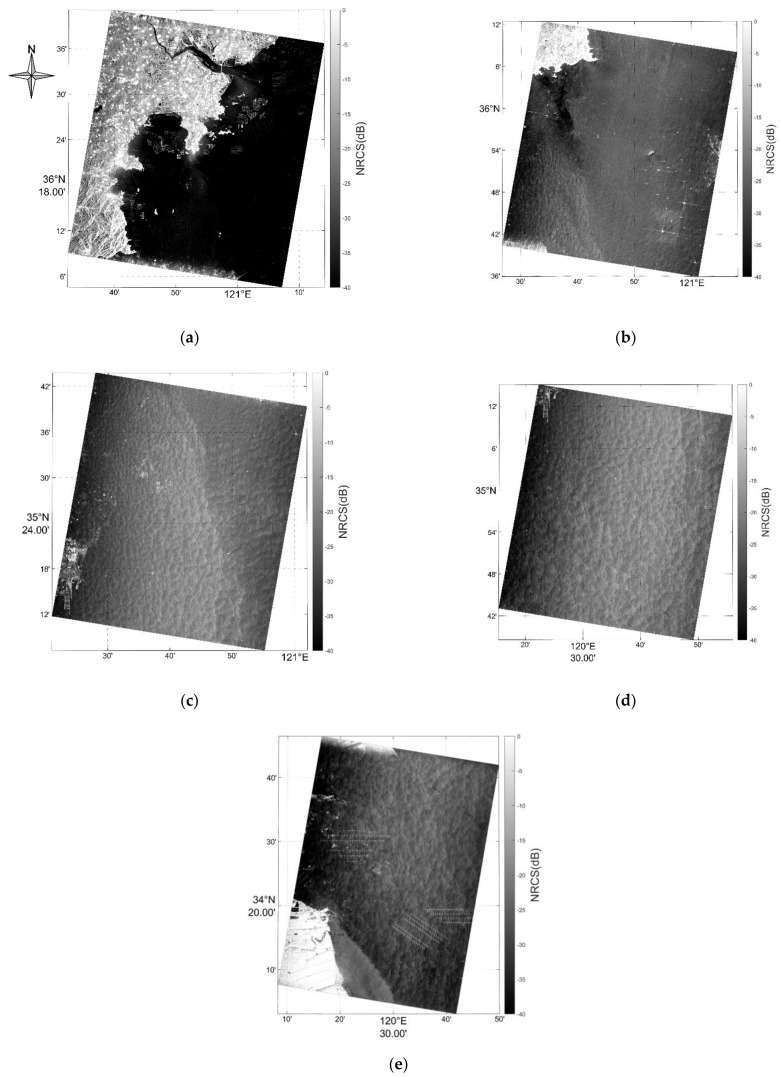
NRCS images of the five GF−3 ATI images acquired in Qing Dao. (**a**–**e**) are the NRCS of region a to region b in Figure 1.

Figure 7 shows the five NRCS images obtained in the experiment conducted in Qing Dao, corresponding to Table 2 in chronological order. It should be noted that these NRCS images were processed using the forward channel data. Image (a)~(d) shows a sea scene in Huang Hai, where ships and their wakes are clear; image (e) shows a seacoast scene nearby Lian Yun Gang.

### 6.1. Result of Data Processing

**Figure 8:**
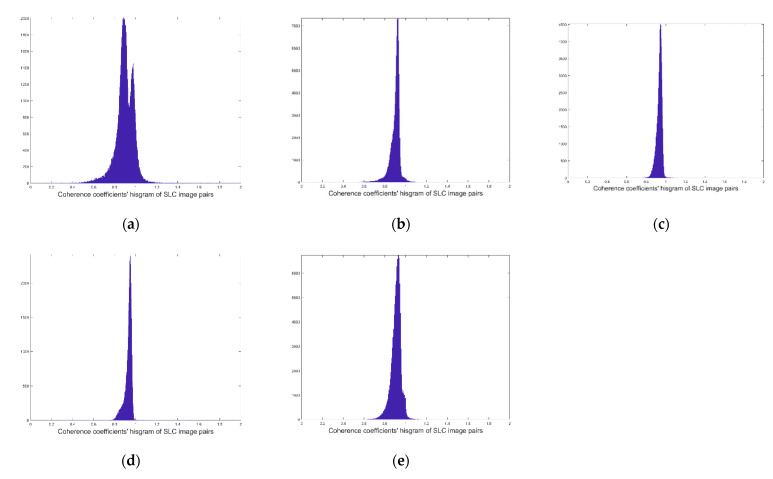
(**a**–**e**) are the coherence coefficients of the SLC image pairs of the five scenes after registration in the Figure 1.

After image registration and correction, the correlation coefficients of the five groups of SAR data are illustrated in Figure 8.

**Figure 9:**
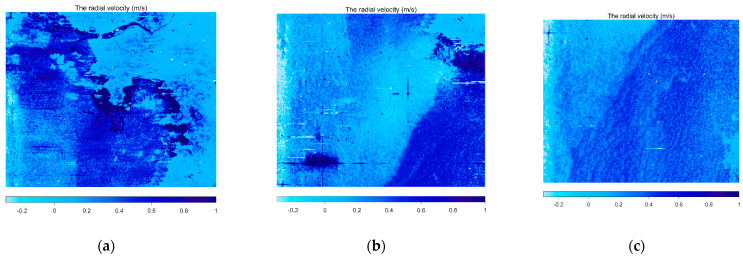

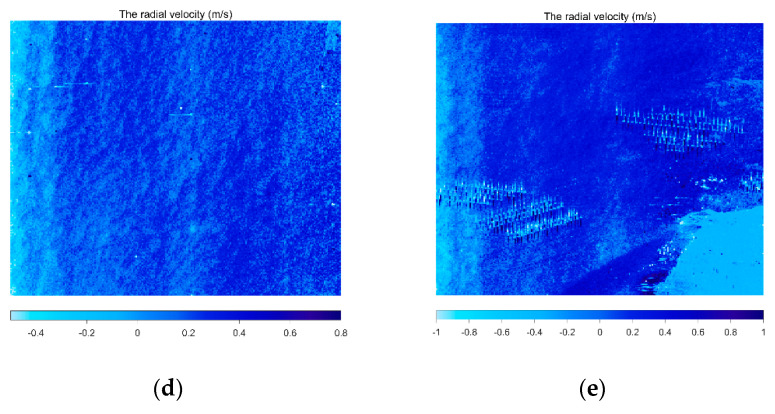
Retrieved radial velocity of SAR images. (**a**–**e**) are the Retrieved radial velocity from the five scenes in the Figure 1.

From Equation (7), the radial velocity $v_{r}$ can be obtained by processing the interference phase of the SAR SLC image. Information on the data processing is presented in Table 3. The coherence coefficients of the SLC image pairs centered on 0.8 to 0.9 after the registration. The calculated radial velocity $v_{r}$ in each SLC image is shown in Figure 9.

### 6.2. Using CDOP Model Retrieval of the Doppler Anomaly

**Figure 10:**
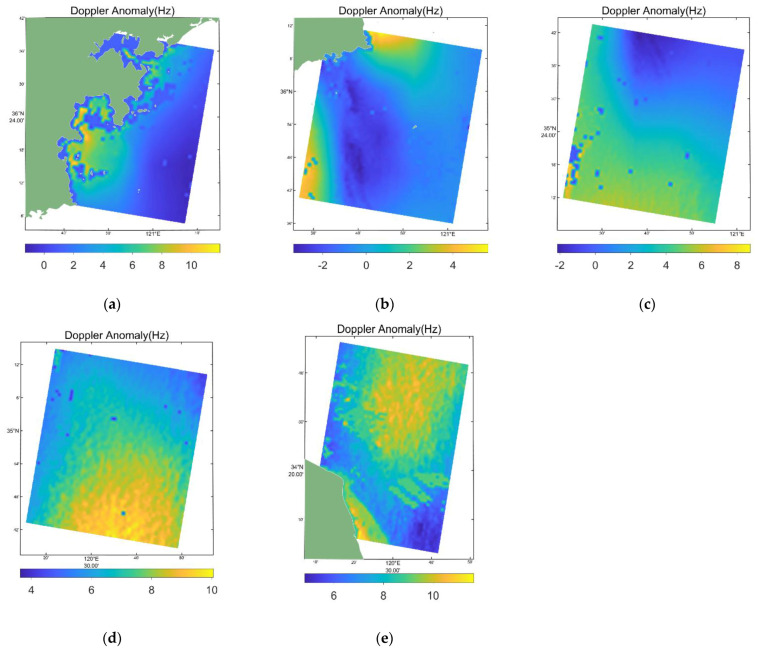
Results of retrieving the Doppler anomaly $f^{DA}$ using the CDOP model. (**a**–**e**) are retrieving the Doppler anomaly results by using the CDOP model from the five scenes in the Figure 1.

Since Bragg waves and large-scale waves are regarded as wind field modulation results, the CDOP model was used to retrieve the Doppler anomaly $f^{DA}$, and the results are shown in Figure 10.

### 6.3. Result of Retrieving the Sea Surface Current

**Figure 11:**
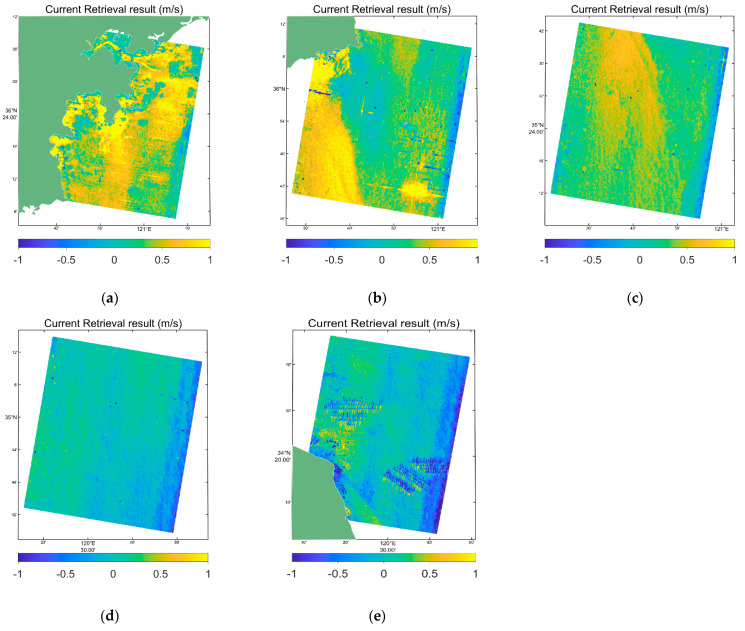
Results of retrieving the ocean surface currents. (**a**–**e**) are retrieving the ocean surface currents from the five scenes in the Figure 1.

In this part, the CDOP model results were used to correct the radial current field in Figure 9. To convert an ocean surface current, the radial velocity should be divided by $\sin\theta_{inc}$, where $\theta_{inc}$ is the incident angle of the radar electromagnetic wave. The retrieval currents of scenes in Figure 7 are shown in Figure 11.

### 6.4. Accuracy Analysis

The retrieval current of images (c) and (d) in Figure 7 was compared with the HFSWR data, because SAR and HFSWR have the same coverage area, as shown in Figure 1. The results are given in Table 4.

The regression coefficients were 0.3931 and 0.5861, respectively, indicating that the retrieved current had a linear relationship with the HFSWR data. It should be noted that the HFSWR is an average result for a sampling interval of 20 min, while SAR is a transient result. Therefore, there are some differences between them, and the correlation is not so strong. In contrast, the current field obtained using wind field correction based on SAR retrieval is closer to the result obtained by the shore-based radar than that obtained using ECMWF correction.

As Table 4 shows, for image (b) and image (c), the correlation coefficients between the retrieved current data and the retrieved wind field and the HFSWR data are 0.4412 and 0.0129, respectively. In Table 5, for image (b) and image (c), the correlation coefficients between the retrieved current data with the ECWMF wind field and the HFSWR data are 0.4593 and 0.0191, respectively. The mean difference between the retrieved current and the HFSWR-derived current is 0.048 m/s and 0.0307 m/s, while the RMSE is 0.2768 m/s and 0.1495 m/s, respectively. In contrast to Table 4, the mean difference between the retrieving current and the HFSWR-derived currents is 0.3047 m/s and 0.3855 m/s, while the RMSE is 0.4161 m/s and 0.4141 m/s in Table 5. Through comparison of the above results, it can be seen that for the same image, the RMSE in Table 4 is smaller, so the corresponding result is better.

**Figure 12:**
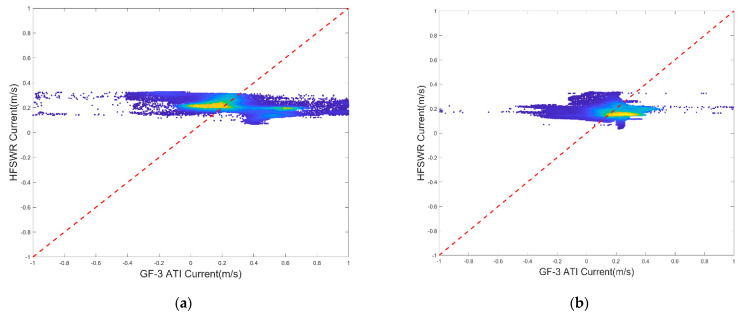
Scatter diagrams of the GF−3 ATI currents processed with the retrieved wind field versus the HFSWR data for the two scenes: (**a**) represents scene b; (**b**) represents scene c.

**Figure 13:**
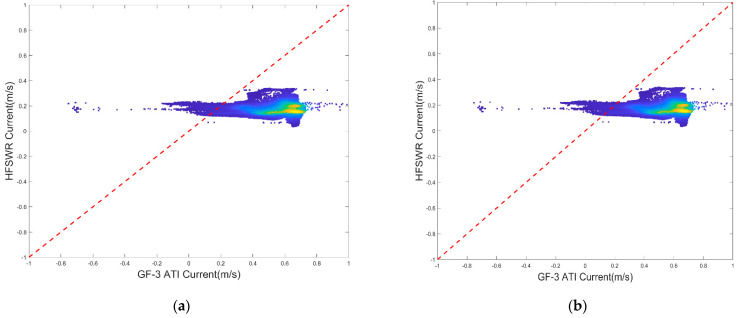
Scatter diagrams of GF−3 ATI currents processed with the ECWMF wind field versus the HFSWR data for the two scenes: (**a**) represents scene b; (**b**) represents scenes c in the Figure 1.

In addition, the consistency of the two results was also investigated. Figure 12 shows the statistical results of the retrieved current, with the retrieved wind field versus the HFSWR data; Figure 13 shows the statistical results of the retrieved current with the ECMWF field versus the HFSWR data. Obviously, there is a good general agreement between the retrieved current and the retrieved wind field and the HFSWR data.

Additionally, for the ECMWF wind field, the CDOP model was used to correct the interference term in the ATI Doppler velocity, and the ocean surface current field was obtained. Scatter diagrams and the statistical results are shown in Figure 13. Compared with Figure 13, the result of currents processed with the retrieved wind field was better than that obtained using the ECMWF reanalysis wind field.

## 7. Conclusions

ATI will be an important approach for observing the sea surface current field in the future. The Doppler velocity measured by ATI includes not only the sea surface current field but also complex interference factors, such as the wind-driven current, sea waves, and Bragg wave phase velocity. Removing these interference factors is the key to retrieving the ATI ocean current field. These interference factors are mainly related to the wind field. In previous studies, external wind field data sources (such as ECWMF) were used, and M4S and other models were employed to calculate and correct the interference factors. However, these external wind field data sources are often the average results of a large range in time and space, while the data obtained by SAR are second-level transient images, which do not match each other. In this paper, the wind field based on SAR data retrieval was used as parameter input, and the CDOP model was adopted to obtain interference factors to correct the ATI Doppler velocity. The experimental results show that the corrected current field results based on the wind field retrieved from SAR data were closer to those obtained by the shore-based radar than those obtained using ECWMF wind field data.

A sea surface current observation experiment in the ATI experimental mode of the GF−3 satellite was performed in Qingdao, China, on 29 January 2022. The one-dimensional sea surface current field was extracted based on satellite data. The accuracy of the surface current inversion in the Qingdao sea obtained on 30 January 2022 was evaluated by comparing it with the HFSWR data. The statistics of the two flows showed good agreement. In our study, two input wind fields were considered, and the experimental results indicated that the GF−3 ATI current processed by the retrieved wind field agreed well with the HFSWR data. The wind field correction result based on the SAR retrieved wind field was closer to the current field measured by the shore-based HF radar than the wind field correction result obtained based on the ECMWF wind field.

The wind field was input into the CDOP model, and then the Doppler anomaly output from this model was subtracted. The research method used in this paper is feasible in theory, but it still has some defects. First, in the phase error correction, only some estimation processing is conducted, and its accuracy is limited. In the ATI-SAR system, phase error can be caused by the antenna phase center error, antenna pointing error, satellite platform flight attitude error, etc. There are also errors in the received echo data. For the verification data (HFSWR data), due to the difference in the working system, the existing measurement methods, and data processing methods, errors inevitably appear in the verification of the oceanic flow field. Therefore, scene error correction of radar measurement remains challenging, and further research on oceanic flow field inversion is needed.

## Figures and Tables

**Table 1:** GF−3 satellite data obtained from the experiment conducted in Qing Dao.

Index	Observation Time (UTC)	Observation Area	Direction	Antenna Direction
a	2022-01-29 21:53:10	120.88° E, 36.38° N	Descending	Right
b	2022-01-29 21:53:18	120.78° E, 35.90° N	Descending	Right
c	2022-01-29 21:53:26	120.69° E, 35.42° N	Descending	Right
d	2022-01-29 21:53:34	120.59° E, 34.95° N	Descending	Right
e	2022-01-29 21:53:43	120.48° E, 34.41° N	Descending	Right

**Table 2:** The radar parameters of the GF−3 satellite.

Radar Parameter	
ATI mode	Full transmission, dual reception
Polarization mode Incident angle Radar operating frequency Pulse repetition frequency	HH, HV 15–50° 5.4 GHz 2202–2606 Hz
Satellite speed	7500 m/s
Effective ATI baseline length	3.75 m
Transmit signal bandwidth	66 MHz
Incident angle	15–50°
Antenna length	15 m
NESZ	−20 dB

**Table 3:** Specification of SLC images.

Image Parameters		
Quantization digit	16 bit	
Image size	Azimuth	23,000 pixel
	Range	16,000 pixel
Spatial resolution	Azimuth	5 m
	Range	5 m
Pixel size	Azimuth	2.59 m
	Range	2.25 m
Image size	Azimuth	60 km
	Range	37 km

**Table 4:** Measured currents processed with the retrieved wind field, and a comparison with GF−3 and HFSWR.

Result	Image (b)	Image (c)
Correlation Coefficient	0.4412	0.0129
Mean Difference	0.0480	0.0307
RMSE	0.2768	0.1495

**Table 5:** Measured currents processed with the ECWMF wind field, and a comparison with GF−3 and HFSWR.

Result	Image (b)	Image (c)
Correlation Coefficient	0.4593	0.0191
Mean Difference	0.3047	0.3855
RMSE	0.4161	0.4141

## Data Availability

Not applicable.

## References

[1] Fischer J., Flemming N.C. (1999). Operational Oceanography: Data Requirements Survey.

[2] Elyouncha A., Eriksson L., Romeiser R., Ulander L. (2019). Measurements of Sea Surface Currents in the Baltic Sea Region Using Spaceborne Along-Track InSAR. IEEE Trans. Geosci. Remote Sens..

[3] Johannessen A., Shuchman R.A., Digranes G., Lyzenga D.R., Wackerman C., Johannessen O.M., Vachon P.W. (1996). Coastal ocean fronts and eddies imaged with ERS 1 synthetic aperture radar. J. Geophys. Res. Ocean..

[4] Romeiser R., Runge H., Suchandt S., Kahle R., Rossi C., Bell P.S. (2013). Quality assessment of surface current fields from TerraSAR-X and TanDEM-X along-track interferometry and Doppler centroid analysis. IEEE Trans. Geosci. Remote Sens..

[5] Goldstein R.M., Zebker H.A. (1987). Interferometric radar measurement of ocean surface currents. Nature.

[6] Thompson D.R., Jensen J.R. (1993). Synthetic aperture radar interferometry applied to ship-generated internal waves in the 1989 Loch Linnhe experiment. J. Geophys. Res. Ocean..

[7] Goldstein R.M., Zebker H.A., Barnett T.P. (1989). Remote sensing of ocean currents. Science.

[8] Romeiser R., Thompson D.R. (2000). Numerical study on the along-track interferometric radar imaging mechanism of oceanic surface currents. IEEE Trans. Geosci. Remote Sens..

[9] Romeiser R., Runge H. (2007). Theoretical evaluation of several possible along-track InSAR modes of TerraSAR-X for ocean current measurements. IEEE Trans. Geosci. Remote Sens..

[10] Toporkov J.V., Perkovic D., Farquharson G., Sletten M.A., Frasier S.J. (2005). Sea surface velocity vector retrieval using dual-beam interferometry: First demonstration. IEEE Trans. Geosci. Remote Sens..

[11] Romeiser R., Breit H., Eineder M., Runge H., Flament P., De Jong K., Vogelzang J. (2005). Current Measurements by SAR Along-Track Interferometry from a Space Shuttle. IEEE Trans. Geosci. Remote Sens..

[12] Romeiser R., Suchandt S., Runge H., Steinbrecher U., Grunler S. (2010). First analysis of TerraSAR-X along-track InSAR-derived current field. IEEE Trans. Geosci. Remote Sens..

[13] Graber H.C., Thompson D.R., Carande R.E. (1996). Ocean surface features and currents measured with synthetic aperture radar interferometry and HF radar. J. Geophys. Res..

[14] Han B., Ding C.B., Zhong L.H., Liu J.Y., Qiu X.L., Hu Y.X., Lei B. (2018). The GF−3 SAR data processor. Sensors.

[15] Wen X.J., Qiu X.L. (2020). Research on Turning Motion Targets and Velocity Estimation in High Resolution Spaceborne SAR. Sensors.

[16] Zhang Q.J. (2017). System Design and Key Technologyies of the GF−3 Satellite. Acta Geod Cartogr Sin..

[17] Frasier S.J., Camps A.J. (2001). Dual-Beam Interferometry for Ocean Surface Current Vector Mapping. IEEE Trans. Geosci. Remote Sens..

[18] Yuan X., Lin M., Han B., Zhao L., Wang W., Sun J., Wang W. (2021). Observing Sea Surface Current by Gaofen-3 Satellite Along-Track Interferometric SAR Experimental Mode. IEEE J. Sel. Top. Appl. Earth Obs. Remote Sens..

[19] Stoffelen A., Verspeek J.A., Vogelzang J., Verhoef A. (2017). The CMOD7 Geophysical Model Function for ASCAT and ERS Wind Retrievals. IEEE J. Sel. Top. Appl. Earth Obs. Remote Sens..

[20] ERA5 Hourly Data on Single Levels from 1959 to Present (Copernicus.eu). https://cds.climate.copernicus.eu/cdsapp#!/dataset/reanalysis-era5-single-levels?tab=form.

[21] Dvornychenko V.N. (1983). Bounds on (deterministic) correlation functions with application to registration. IEEE Trans. Pattern Anal. Mach. Intell..

